# Proniosomal Gel-Loaded Phosphodiesterase Inhibitors (Sildenafil, Vardenafil, and Tadalafil): Prospects for Topical Penile Therapy of Tadalafil for Treatment of Erectile Dysfunction

**DOI:** 10.3390/gels9080597

**Published:** 2023-07-25

**Authors:** Soad A. Mohamed, Remon Roshdy Rofaeil, Hesham Salem, Mahmoud Elrehany, Yahya I. Asiri, Adel Al Fatease, Hamdy Abdelkader

**Affiliations:** 1 Department of Pharmaceutics, Faculty of Pharmacy, Deraya University, New-Minia 61519, Egypt; soad.ali@deraya.edu.eg; 2 Department of Pharmacology, Faculty of Pharmacy, Deraya University, New-Minia 61519, Egypt; remon.roshdy@deraya.edu.eg; 3 Department of Pharmacology, Faculty of Medicine, Minia University, Minia 61511, Egypt; 4 Department of Analytical Chemistry, Faculty of Pharmacy, Deraya University, New-Minia 61519, Egypt; hesham.salem@deraya.edu.eg; 5 Department of Biochemistry, Faculty of Pharmacy, Deraya University, New-Minia 61519, Egypt; mahmoud.elrehany@deraya.edu.eg; 6 Department of Pharmacology, College of Pharmacy, King Khalid University, Abha 62223, Saudi Arabia; yialmuawad@kku.edu.sa; 7 Department of Pharmaceutics, College of Pharmacy, King Khalid University, Abha 62223, Saudi Arabia; afatease@kku.edu.sa

**Keywords:** tadalafil, intromission, permeability, penile therapy, erectile dysfunction, mounting

## Abstract

Oral phosphodiesterase inhibitors have emerged as a game changer for the treatment of erectile dysfunction (ED) since attaining FDA approval for its first member, sildenafil, in 1998. Topical penile therapy could be a viable replacement for oral medication that would transform the treatment of ED for many decades to come. This innovative idea could offer a safer topical alternative with less vision and cardiovascular side effects than the oral route. This work aims at developing proniosomal gels for three selected members (sildenafil, vardenafil, and tadalafil) and investigating the proniosomal gels on a rodent model. Niosomes derived from the parent proniosomal gels were characterized for entrapment efficiency (EE%), size, polydispersity index (PDI), zeta potential, and morphology. Proniosomal gels were evaluated for skin permeation, in vivo mating behaviors, and biochemical assays of nitric oxide (NO) and cyclic guanosine monophosphate (cGMP) post penile topical administrations. The optimized proniosomes loaded with tadalafil (F1-T) were compared with oral tablets (Cialis^®^). Proniosomal gels demonstrated significant enhancement of skin penetration by up to 5.5-fold, compared to control topical suspension. Tadalafil-loaded proniosomes showed superior skin permeability over sildenafil- and vardenafil-loaded proniosomes. In addition, significant improvement was noticed regarding intromission number, intromission ratio, NO, and cGMP for the proniosomal gel F1-T, compared to the untreated control. No statistically significant (*p* > 0.05) differences in sexual performance or biochemical parameters (NO and cGMP levels) were recorded among orally and topically (tadalafil proniosomal gel) administered groups. These findings support tadalafil topical penile therapy as a promising alternative to the oral route.

## 1. Introduction

Erectile dysfunction (ED) is a gender-specific disease that affects men. ED can be defined as insufficient erection for completing satisfactory intercourse. This is a very common (50%) medical problem affecting elderly men at the age of >50 years. Men with preexisting chronic diseases, like diabetes, Parkinson’s disease, renal failure, prostate cancer, and some traumatic pelvic injuries, are at high risk of experiencing ED [1,2]. Current statistics indicate that the prevalence is 5–20% of the adult male population [3]. Grievously, the global prevalence of ED progressively increases; more than 300 million men are likely to be affected worldwide by 2025 [4].

With the launching of the phosphodiesterase 5 (PDE5)-inhibitor prototype sildenafil in the late 1990s and emerging other members, such as vardenafil and tadalafil 10 years later, PDE5 inhibitors took over the first-line treatment as a non-invasive alternative to penile injections (e.g., papaverine and alprostadil injections) [2]. The PDE5 inhibitors inhibit cyclic guanosine monophosphate (cGMP) phosphodiesterase in the corpus cavernosum, resulting in the relaxation of penile smooth muscles, and heighten blood flow that is required for sufficient erection and penetrative intercourse [5].

The PDE5 inhibitors structurally mimic cyclic cGMP; they bind to PDE5 competitively and reduce cGMP breakdown, which leads to vasodilation and increased blood flow, which result in penile erection. The currently approved PDE5 inhibitors are four (sildenafil, tadalafil, avanafil, and vardenafil) with comparable efficacy. Globally, the two most common PDE5s are sildenafil and tadalafil. In 2003, tadalafil was released as a selective, long-acting PDE5. The tadalafil onset of action is 20 min, with a long duration up to 72 h [6,7].

The PDE5 inhibitors revolutionized the treatment modality; this is to say, a single pill that can be simply administered orally and sufficiently provide symptomatic treatment of ED. There are many generic pharmaceutical products; such as sildenafil (Viagra^®^ 50 mg and 100 mg tablets), tadalafil (Cialis^®^ 5 mg and 20 mg tablets), vardenafil (Levitra^®^ 10 mg and 20 mg tablets), and avanafil (Spedra^®^ 100 mg tablets); available as over-the counter medicine dispensed in community pharmacies without prescription in some countries. The gravity of this practice has led to some fatalities. A history of cardiac events and cardiovascular risk could be excerpted effects resulting in death. There have been reports of myocardial infarction, cardiac arrest, and ischemic attacks following the marketing of several PDE5 inhibitors [8,9].

In addition to the medical drawbacks of PDE5 inhibitors, there are several limitations with the current oral therapy. To achieve the desired medical plasma concentrations, it is advised that they be taken either daily or prior to sexual activity. This mode of therapy limits spontaneity, and it has a long half-life that is far longer than typical sexual encounters. Several adverse effects were reported with this mode of administration, such as headache, back pain, and visual disturbances, which lead to discontinuation. Further, preexisting conditions of unstable angina and the use of nitrates contraindicate the use of oral PDE5 inhibitors [2,10].

The search for topical penile therapy for ED has been studied for decades. Topical glyceryl trinitrate (0.2%) gel (DermaSys) was formulated for the treatment of ED. In a clinical investigation on 232 men, the start of erection was recorded within 10 min with 69.5% of all intercourse attempts with MED2005, indicating significant improvement in mild ED patients. However, common side effects reported for both patients and partners included headache (patients 7.9% and partners 1.3%) and nasopharyngitis (patients 5.7% and partners 0.9%) [10]. Topical alprostadil cream containing increasing doses of 50, 100, 200, and 300 µg per dose was applied to the tip of the penis using a single-unit dose dispenser of 100 mg cream. Dose-related efficacy was observed and limited to the 100, 200, and 300 µg doses. Localized adverse effects (genital pain, tenderness, and erythema) were recorded for a short time [11]. These findings suggest that topical delivery could be a viable option in the treatment of ED.

Proniosomes can be defined as niosome concentrates that have a gel texture due to minimal water content. This is an approach that has been commonly researched as a suitable vehicle for skin and transdermal delivery of many therapeutic drugs [12,13]. Proniosomes have more potential advantages over niosomes and liposomes. Proniosomes have a gel-like structure; they are more suitable for application on skin. Proniosomes do not need the addition of gelling agents like other vesicular systems [14]; proniosomes are less leaky for entrapped drugs than niosomes [12]. They can transform into niosomes at the application site (e.g., skin) under occlusive conditions [13]. Proniosomes are dominantly composed of surfactant forming; such as Span 20 (sorbitan monolaurate), Span 40 (sorbitan monopalmitate), Span 60 (sorbitan stearate), and Span 80 (sorbitan oleate); and membrane stabilizers, such as lecithin and cholesterol. The coacervation-phase separation method is one of the most commonly used techniques to generate proniosomal gels [13]. Surfactants and lipids (cholesterol and lecithin) are dissolved in hot alcohol above the phase-transition temperature of the surfactants (60 to 70 °C). The pre-warmed aqueous phase is added at the same temperature, the whole mixture is agitated/vortexed, it finally is allowed to cool, and a proniosomal gel is then harvested.

The goal of this study was to create proniosomal gels containing the three PDE5 inhibitors sildenafil, tadalafil, and vardenafil. The optimized formulation was topically applied to study the mating behavior in a rodent model. Specific objectives looked into studying the two lipid membrane stabilizers Compritol 888 ATO (glyceryl dibehenate) and Precirol ATO 5 (glyceryl distearate), particle sizes, morphology, and skin permeability. The mating behavior and sexual activities were measured through the estimation of various parameters, such as mounting number, intromission number, intromission ratio, mounting latency, and intromission latency. Further, the biochemical determination of nitric oxide and cGMP in penile tissue was also studied and compared with orally administered tadalafil.

## 2. Results and Discussion

Nine different proniosomal gels were prepared using the coacervation-phase separation method. Three proniosomal gels (F1–F3) of different compositions were prepared for each drug. The three proniosomal formulations were composed of Span 60 (surfactant forming proniosomes), membrane bilayer stabilizers (cholesterol and lecithin), and two different lipidic materials (Compritol 888 ATO and Precirol ATO 5). Compritol and Precirol are multipurpose lipid excipients and are commonly used in pharmaceutical and cosmetic industries as emulsifying agents, viscosity enhancing agents, and sustained-release forming lipids [15]. Pluronic F127 at a concentration of 0.01% was employed in this study as a permeation enhancer. Preformulation studies indicated a promising role for Pluronic F127 as a skin-penetration enhancer [16]. The nine different proniosomal gels were characterized for entrapment efficiency (EE%), size, polydispersity index (PDI), zeta potential, morphology using the scanning electron microscope (SEM), and permeation through excised rat skin.

### 2.1. EE% and Drug Loading

The EE% recorded for the prepared proniosomal gels was in the range of 45% to 97% (Table 1). This wide and significant (*p* < 005) difference in EE% was likely due to possible variables. The composition of proniosomes and the drug type entrapped in proniosomes were evident to be the two significant players affecting EE%. A minimum amount of cholesterol was employed at a molar ratio of 7:3 for surfactant: cholesterol ratio. It has been reported that a minimum amount of cholesterol is in favor of EE% [17,18]. This could explain the high EE% (93% to 97%) recorded for F1 irrespective of drug type. Higher cholesterol levels > 30% have been associated with lower EE% due to increased rigidity of the bilayer membranes of niosomal vesicles, and this reduces their accommodating ability as cargo for drug loading [19]. Compritol- and Precirol-containing proniosomes (F2 and F3, respectively) showed significantly lower EE% than those from F1. Compritol (glyceryl dibehenate) and Precirol (glycerylpalmitostearate) are extremely lipophilic lipids (HLB = 2) with saturated hydrocarbon chains and high transition melting points (60 to 70 °C) [15,20]. These properties might undermine the loading capacity of the generated proniosomes. With regard to the effect of cargo load on EE%, tadalafil-loaded proniosomes (F1-T to F3-T) demonstrated superior EE%, compared to both vardenafil- and sildenafil-loaded proniosomes. On the contrary, sildenafil-loaded proniosomes (F1-S to F3-S) showed the lowest EE% irrespective of the composition. While there are no marked differences in the molecular weights (389.4, 488.6, and 474.6, respectively) of tadalafil, vardenafil, and sildenafil, the partition coefficient (log *p*) values recorded marked differences (0.4, 1.8, and 3, respectively) for sildenafil, vardenafil, and tadalafil [16]. A higher partition coefficient indicates greater lipophilicity; hence, the drug is more favorable to be accommodated in surfactant/lipid bilayer and is less likely to leak into the aqueous domain. It is widely accepted that lipophilic drugs record high EE% and more-hydrophilic drugs shows low EE% of vesicular systems, such as niosomes, liposomes, and proniosomes [19].

### 2.2. Size, PDI, and Zeta Potential

The average sizes for niosomes derived from proniosomes were in the range of 1.81 to 4.5 µm (Table 1). The main variable that contributed to significant differences in particle size was the composition of the prepared proniosomes. On contrary to EE%, the effects of drug types on proniosomal sizes showed no-to-insignificant differences. For example, F1-S, F1-V, and F1-T recorded average sizes of 1.89, 1.81, and 1.80 µm, respectively. Similarly, F3-S, F3-V, and F3-T registered an average size of 4.5 µm. On the other hand, the composition had a significant role in determining the proniosomal sizes. Irrespective of cargo load, Span 60 proniosomes (F1) showed significantly smaller sizes (approximately 1.8 µm) than that (4.5 µm) recorded for Span 60/Precirol-containing proniosomes (F3) [21,22].

The polydispersity index (PDI) recorded for the prepared proniosomes ranged from 0.19 to 0.39, indicating a narrow size distribution for the prepared proniosomes. The zeta-potential values recorded were in the range of −37.7 to −43.9 mV. High surface charge indicates an acceptable physical stability and less likelihood to aggregate [21].

### 2.3. Morphology

**Figure 1:**
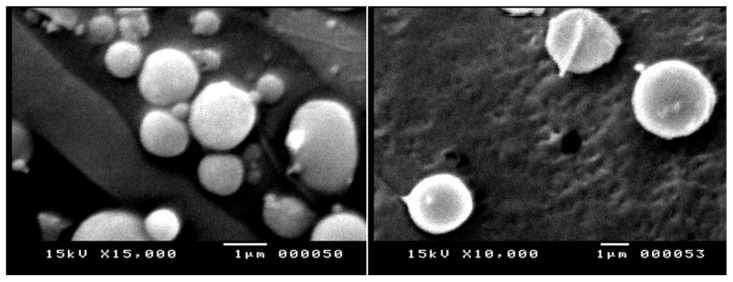
SEM micrographs for niosomes derived from proniosomal gel (F1-T).

Morphology and surface characteristics were studied for some selected niosomes derived from proniosomes (F1). They had smooth and spherical vesicles with an average size range of >1 µm with narrow size distribution (Figure 1). These results correlated well with those obtained from the dynamic-light-scattering technique using the Nanosizer.

### 2.4. In Vitro Release Studies and Kinetics of Release

**Figure 2:**
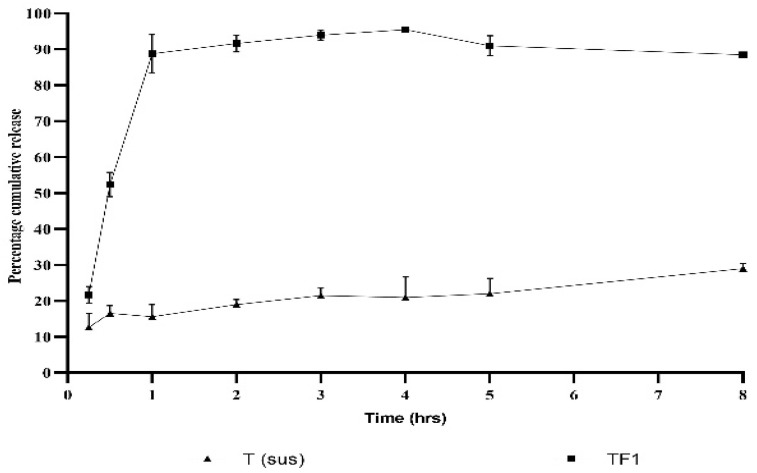
In vitro release of tadalafil F1-T from proniosomal gel compared to tadalafil suspension over 8 h.

A substantial release of the tadalafil from F1-T proniosomal gel was observed within 30 min of processing, which increased to 89% in 1 h and continued at these high values for 8 h without a tendency to decline. On the other hand, when the release of tadalafil from its suspension in PBS 6.7 ± 0.5 was observed, only 20% of the compound released after 4 h (Figure 2). Regarding kinetics, the release of tadalafil from F1-T proniosomal gel follows the Higuchi model (r = 0.95), which indicates a controlled release of tadalafil, as illustrated in Table 2.

### 2.5. Ex Vivo Skin Permeation Studies

**Figure 3:**
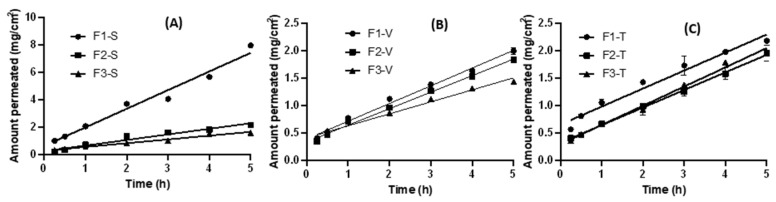
Ex vivo permeation profiles from sildenafil—(**A**), vardenafil—(**B**), and tadalafil-loaded proniosomes (**C**) using the excised rat skin. Data points represent mean ± SD.

Drug permeability from the prepared proniosomes through the excised skin rat was studied, and permeation profiles for the three drugs from proniosomes are shown in Figure 3A–C. Two permeation parameters (flux and apparent permeability (P_app_)) were calculated and are shown in Table 1.

It is evident that the permeation parameters were significantly dependent on the composition of the proniosomes and drug. Irrespective of the drug loaded in the proniosomes, F1 showed superior permeability. For example, F1-S, F2-S, and F3-S recorded flux and P_app_ values of 0.77 mgh^−1^·cm^−2^ and 77.0 cm·h^−1^, 0.23 mg·h^−1^·cm^−2^ and 23.0 cm·h^−1^, and 0.17 mg·h^−1^·cm^−2^ and 17.0 cm·h^−1^, respectively. This significant enhancement of the apparent permeability of F1-S, compared to F2-S and F3-S by 3.3- and 4.5-fold, respectively, could be mainly attributed to a couple of reasons: Firstly, F1 and F2 recorded significantly smaller vesicular sizes compared to F3, and therefore, F1-S and F2-S had larger surface areas and better enhancing permeation of sildenafil. Secondly, Compritol 888 ATO and Precirol ATO 5 have higher solid–liquid transition temperatures (66–73 °C); therefore, the generated F2 and F3 niosomes were in relatively greater gel states, resulting in reduced partitioning of the cargo load out of the vesicles [19]. It is worth mentioning that all niosomes derived from proniosomes regardless of whether the composition significantly (*p* < 0.05) improved skin permeability, compared to that from drug suspensions reported in our previous study [16]. For example, previously published data showed apparent permeability for sildenafil suspension (P_app_ = 14.0 cm·h^−1^) and sildenafil suspension containing 0.01% Pluronic F127 (P_app_ = 24.0 cm·h^−1^) [16], which were significantly (*p* < 0.05) less than that (P_app_ = 77.0 cm·h^−1^) recorded for F1-S in the current study. The permeability enhancement factors for F1-S recorded 5.5- and 3.2-fold, compared to the sildenafil suspension and sildenafil suspension, respectively, containing 0.01% Pluronic F127. Proniosomes are surfactant-based vesicles in which the drug molecules exist in a soluble form ready for partitioning, larger surface area, improved rheological properties for better retention at the site of application, and greater spreading ability [12,23]. Apparent permeability (P_app_) of tadalafil-loaded proniosomes (F1-T) recorded the greatest value of 125 cm·h^−1^, while sildenafil-loaded proniosomes (F1-S) showed the lowest P_app_ = 77.0 cm·h^−1^ and vardenafil-loaded proniosomes came in the middle with P_app_ = 90.0 cm·h^−1^.

The log *p*-values reported for sildenafil, vardenafil, and tadalafil are 0.4, 1.8, and 3.0, respectively [16]. The higher the log *p*-values, the better lipophilicity and greater permeability of the drug to cross skin lipid barriers. This could explain the superior permeability of F1-T, compared to both F1-S and F1-V. Therefore, F1-T was selected for the in vivo studies due to superior permeability.

### 2.6. Mating Behavior Studies and Sexual-Parameter Estimation

**Figure 4:**
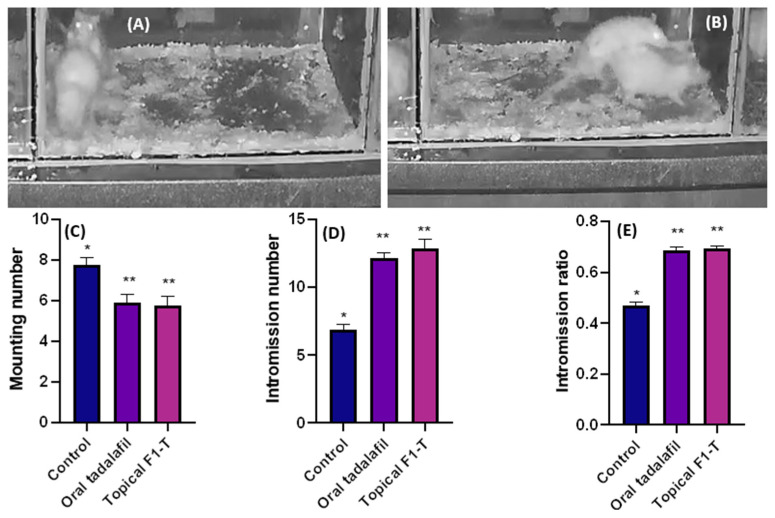
Sexual-activities video recorded for mounting (**A**) and intromission (**B**); sexual parameters measured as mounting number (**C**), intromission number (**D**), and intromission ratio (**E**) for the control (untreated group) and groups orally treated and topically treated with tadalafil. Data were expressed as mean ± SD, n = 8. The * denotes statistically significant differences (*p* > 0.05) from the control group, and ** denotes statistically non-significant differences (*p* < 0.05) between treated groups.

Three groups (untreated control, oral tadalafil, and topical F1-T) were studied for sexual activities. The mating animals were video reordered, and the numbers of mountings and intromissions were captured, as shown in Figure 4A,B. These mating behaviors were counted for mountings and intromissions (Figure 4C,D). The intromission ratio (Figure 4E) was estimated, as it is an indicative parameter that can measure the sexual potency and capacity [24].

These parameters were employed to assess the sexual activities; the mounting behavior does not necessarily end up with intromission and ejaculation. On the contrary, the intromission behavior required sufficient erection for penetration of the receptive female animal [24,25]. Mounting behavior has been described as failed intromission due to insufficient erection [26]. Sexual stimulation can happen because the male rats continue to investigate the estrous female partners. An increasing number of mounting attempts indicated abortive intromission due to erectile dysfunction [27]. This could explain the significant reduction in the number of mountings for oral (58) and topical tadalafil (60) compared to that (80) for the untreated group (Figure 4C).

**Figure 5:**
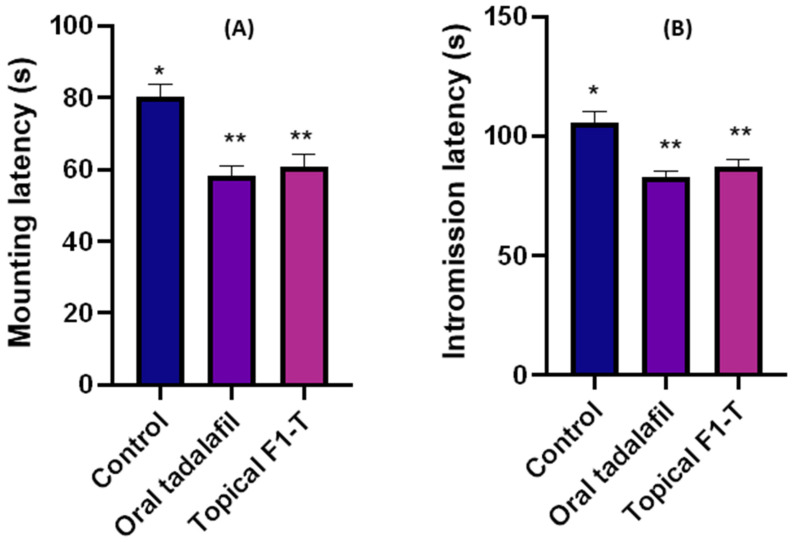
Mounting latency (**A**) and intromission latency (**B**) for the control (untreated group) and groups orally treated and topically treated with tadalafil. Results are expressed as mean ± SD, n = 8. The * denotes a statistically significant difference (*p* > 0.05) from the control group, and the ** denotes a statistically non-significant (*p* < 0.05) difference between the treated groups.

On the other hand, the intromission ratio for the control was 0.46, while the intromission ratios recorded for the oral and topical tadalafil groups were 0.68 and 0.69, respectively. These significant (*p* < 0.05) increases in the intromission ratios for the tadalafil-treated groups indicated the increase in sexual potency and erection. More interestingly, there were no statistically significant differences among the rats who received oral or topical treatment with the same drug. This suggested that the topical route is equally effective as the oral route. Latency time for mounting and intromission significantly (*p* < 0.05) decreased compared to that for control (Figure 5A,B). This indicated an increase in both sexual activities and potency [27].

When compared to the control group, topical application of F1-T increased both the intromission number and ratio significantly (*p* < 0.05). At the same time, there was no discernible difference between oral tadalafil and topically applied proniosome-loaded tadalafil (F1-T).

Moreover, oral and topical applications of the formulation significantly reduced the mounting number and latency time for both mounting and intromission, compared to the control group. No significant (*p* > 0.05) changes in mounting number or latency time occurred between oral and topical tadalafil.

Tadalafil improves the patient’s sexual confidence directly through improving erectile function and indirectly through increasing spontaneity and ameliorating time concerns or latency periods [28,29]. This could explain the significant increase in the intromission frequency and latency period for both oral and topical administration. More interestingly, the topically applied formulations achieved a similar efficiency to the oral group.

### 2.7. Biochemical Assays for the Excised and Homogenized Penile Tissues

Nitric oxide (NO) is thought to play an essential role in erection. Nitric oxide activates guanylate cyclase, which, in turn, initiates the formation of cGMP that relaxes cavernous smooth muscle, a requirement for the start and support of erection [30].

**Figure 6:**
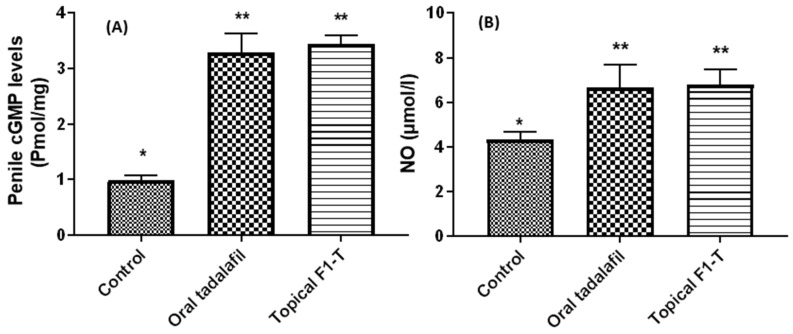
Biochemical parameters cGMP (**A**) and NO (**B**) measured in the excised and homogenized penile tissues measured for the control (untreated group) and groups orally treated and topically treated with tadalafil. Data were expressed as mean ± SD, n = 8. The * denotes statistically significant differences (*p* > 0.05) from the control group, and the ** denotes statistically non-significant differences (*p* < 0.05) between treated groups.

The male organs for the three groups were excised and homogenized for quantitation of cGMP and NO levels. Both cGMP and NO levels were estimated for two reasons: firstly, due to their role in initiating and maintaining erection and, secondly, to confirm our hypothesis that tadalafil can exert its effects topically as potently as orally (Figure 6). The results demonstrated that both cGMP and NO levels were significantly (*p* < 0.05) increased, compared to the untreated control group. Further, there were no statistically significant differences in cGMP and NO levels between orally and topically treated male rats with tadalafil. This indicated that both oral and topical tadalafil induced a significant increase in penile NO and cGMP. Such an increase was reflected in the enhancement of initiation and maintenance of penile erection.

## 3. Conclusions

This study investigated the hypothesis of topical penile therapy of three widely known phosphodiesterase inhibitors: sildenafil, vardenafil, and tadalafil. These three drugs were loaded into Span 60-based proniosomal gels of different compositions. Two additional lipids, Compritol and Precirol, were investigated. Proniosomal gels demonstrated advanced skin permeability compared to topical control suspension formulations by 5.5 increments. Tadalafil showed superior flux and enhanced skin permeability by up to 1.6 compared to sildenafil- and vardenafil-loaded proniosomal gels, compared to sildenafil and vardenafil. Mating behavior and biochemical assays of the two biochemical parameters involved in erection (NO and cGMP) were evaluated. Sexual behaviors were well documented through in-house night-vision camera recording. Undoubtedly, topical penile therapy of tadalafil utilizing the proniosomal system significantly enhanced sexual activities, indicated by NO and cGMP levels, compared to the untreated group. This clinical study holds a promise for tadalafil topical penile treatment of erectile dysfunction, which might help many individuals when systemic phosphodiesterase-inhibitor medication is contraindicated.

## 4. Materials and Methods

Sildenafil citrate, vardenafil, and tadalafil were kindly supplied by Pharco Corporation, Alexandria, Egypt. Lecithin and Pluronic F127 were bought from Sigma Aldrich (St. Louis, MO, USA). Span 60 was bought from Oxford Lab Chem, Mumbai, India. Comparitol 888 ATO and Precirol ATO 5 were a kind gift from Gattefosse Corporation, Lyon, France.

### 4.1. Methods

#### Preparation of Proniosomes

Sildenafil, tadalafil, and vardenafil proniosomes were prepared based on the method mentioned elsewhere [31]. In brief, the drug, Span 60, lecithin, and cholesterol were mixed alternatively with Compritol or Precirol and all dissolved in 1 mL of absolute ethanol in a tightly closed glass vial and heated in a water bath for 5 min at 70 ± 1 °C.

The aqueous phase was composed of Pluronic F127 (0.02%) in distilled water (1 mL). The temperature of the aqueous phase was raised to 70 °C. The aqueous phase was transferred into the organic phase and heated for an extra 5 min until a clear mixture was obtained. The final solution was allowed to cool until the proniosomal dispersions formed as creamy viscous gels of niosome concentrates. The composition of the prepared proniosomal gels is shown in Table 3.

### 4.2. Encapsulation Efficiency (EE%)

The EE% calculations of the prepared proniosomes were assisted using the centrifugation method. The proniosomes were diluted by adding 2 mL of prewarmed distilled water and vortexed for 2 min to form niosomal dispersions. The final niosomal dispersions were centrifuged at 13,000 rpm for 30 min at 4 °C. The supernatant was discarded, and the pellets were redispersed in 2 mL of distilled water. The free unentrapped drug was analyzed for sildenafil, tadalafil, and vardenafil using an ultraviolet (UV) spectroscopic method by recording the absorbance at 292, 285, and 272 nm, respectively, using a UV–visible spectrophotometer (Shimadzu, Koyoto, Japan). The EE% was calculated through Equation (1).

(1) $$\mathrm{EE}\%=\frac{X_{t}-X_{f}}{X_{t}}\times 100$$

where *X_t_* and *X_f_* denote total amount of drug and free unentrapped drug, respectively.

### 4.3. Size and Zeta-Potential Measurements

The Z-average diameter and zeta potential were measured using the Zeta sizer (Malvern analytical Ltd., Malvern, UK). The proniosomal gels were diluted with 10 mL of deionized water prior to carrying out measurements.

### 4.4. Scanning-Electron-Microscope Imaging of Proniosomal Formulations

The prepared proniosomal gels were diluted by adding 20 mL of distilled water. One drop was added on an aluminum stub. The niosomes formed were then sputter-coated with gold/palladium under vacuum and visualized using a scanning electron microscope (KYKY EM 3200 digital scanning electron microscope, Beijing, China) at 25 kV of accelerating voltage.

### 4.5. In Vitro Release and Dissolution Kinetics of the Free-Tadalafil and Optimum-Tadalafil Proniosomal Gel (F1T)

The in-vitro release of tadalafil from the proniosomal gel was performed using the dialysis membrane method [27]. An amount of 10 mg of F-1T proniosomal gel and a 4 mg equivalent of the entrapped pure drug were transferred to two dialysis bags with a molecular-mass cutoff of 12,000 Da. The bags were suspended in 20 mL of ethanol:phosphate buffer saline (PBS, PH = 7.2) with a ratio of 3:7 (*v*/*v*) at 37 °C ± 0.5 °C in a shaking water bath (JSSB-Series Water Bath, Shandong, China) at 100 rpm. At designated time intervals (0.5, 1, 2, 4, 6, and 8), 0.5 mL of the blank medium was added into the receptor compartment to keep the volume constant. The mechanism of release was determined by the following kinetic models.

Zero-order: 𝑅 = 𝐾_0_𝑡 

First-order: $R=1-e^{-k1}t$

Higuchi diffusion model: $Q=K_{H}\cdot t^{1/2}$

Baker–Lonsdale model: $3⁄2[1-{\left(1-M_{t}⁄M_{\infty }\right)}^{2/3}]M_{t}⁄M_{\infty }=K_{3}t$

Hixson–Crowell cube-root law: ${UR}^{1/3}=k_{4}t$

### 4.6. Ex Vivo Permeability

Drug permeability from the proniosomal gels was studied using a freshly excised full-thickness dorsal rat skin mounted on a donor compartment of in-house-modified Franz diffusion cells, as previously mentioned [16]. Accurately measured amounts of proniosomal gels, containing 10 mg, 2 mg, and 2 mg of sildenafil, tadalafil, and vardenafil, respectively, were used. The receptor compartment was filled with 25 mL of citrate buffer of pH 5.5, and the temperature was adjusted to 32 °C ± 1 °C using a shaking water bath (Köttermann, Hänigsen, Germany) at 100 strokes per minute. A sample of 2 mL was collected at predetermined time points. The samples were determined using UV spectrophotometry, as mentioned above. The permeation flux and apparent permeability coefficient were determined as reported in our previous publication [16].

### 4.7. In Vivo Studies

#### 4.7.1. Animals and Grouping

Sprague Dawley male rats were used as a well-representative animal model for studying ED [32]. Thirty Sprague Dawley male rats (200–225 gm) and 36 Sprague Dawley female rats (200–220 gm) were employed in excess numbers. The animal ethical committee at the Faculty of Pharmacy, Deraya University, approved the procedures with animals (approval no. 3/2022). The male rats were screened for their sexual activity for 7 days before the experiment. Those males that showed no intromission within 30 min in the test were excluded due to sexual inactivity [24]. The male rats that passed the screening test were divided into three groups (8 rats per group):

Group I: Control group (untreated).

Group II: Oral tadalafil group received an oral dose of tadalafil tablets (Cialis^®^) = 10 mg/kg suspended in 0.5% carboxymethylcellulose solution via a gastric lavage tube [33].

Group III: Topical group received a topical dose (100 µL) of F1-T proniosomal gel = 10 mg/mL applied on the shaft of the penis and glans.

The female rats were presented at the estrus phase through a daily subcutaneous injection (1 mg/kg/d estradiol benzoate) for 5 days [5]. Receptive female rats were confirmed by exhibiting the lordosis posture during mounting. If the female rat did not exhibit the lordosis behavior, the animal was replaced [24,34].

#### 4.7.2. Mating Behavior Assay

The mating animals were kept in a dark room for one hour in a transparent glass box (60 × 60 × 80 cm). The experiment was performed in the early morning (1:00 AM to 5:00 AM). The mating behaviors were recorded using a night-vision camera (Hikvision network camera, Hikvision Digital Technology, Co., Ltd., Hangzhou, China) and the iVMS V3.9.0.5 software (Hikvision, IBCAM, Hangzhou, China). The mounting number, intromission number, mounting latency, intromission latency, and intromission ratio were the sexual behavioral parameters recorded [35,36].

Equation (2) was used to calculate the intromission ratio:

(2) $$\mathrm{Intromission}\;\mathrm{ratio}=\frac{In}{Mn+In}$$

where In and Mn are the numbers of intromissions and mountings, respectively.

### 4.8. Biochemical Tests

#### 4.8.1. Nitric Oxide (NO)

The level of NO was estimated in the penile tissues after the mating behavior test using the Griess reagent [37]. Flash-frozen tissues with liquid nitrogen were homogenized in 10 volumes of 0.1 M PBS, pH 7.2, then centrifuged to obtain the supernatant to apply for analysis. The NO kit was supplied by Biodiagnostic, Cairo, Egypt. In brief, the samples rendered acidic and were diazotized by sulphanilamide and then coupled with N-naphthyl ethylenediamine, resulting in a stable azo dye measured at 540 nm.

#### 4.8.2. Cyclic Guanosine Monophosphate (cGMP)

The penile tissues were flash frozen in liquid nitrogen, grinded in a small mortar, and homogenized in 0.1 M HCl (10 mL). The homogenate was centrifuged at 600× *g* to pellet the debris. The supernatant was used for analysis [38]. The cGMP concentrations were determined with the ELISA cGMP detection kit (Abcam, ab133052, Boston, MA, USA).

### 4.9. Statistical Analysis

GraphPad Prism 8.4.3 was used for statistical analysis of the results, which included Student’s *t*-test to compare two groups and a two-way ANOVA to compare groups (686). http://www.graphpad.com. Accessed 25 June 2022.

## Figures and Tables

**Table 1:** Physicochemical properties (size, polydispersity index, and zeta potential) and permeation parameters (flux and apparent permeability coefficient) for the prepared proniosomes. Data represent mean ± standard deviation (SD).

Formulation	EE%	Drug Loading Capacity (%)	Particle Size (µm)	PDI	Zeta Potential (mV)	Flux (mg·h^−1^ cm^−2^)	Apparent Permeability P_app_·10^−3^ (cm h^−1^)
F1-S	93 ± 2.0	0.62	1.89 ± 0.10	0.29 ± 0.01	−43.9 ± 2.5	0.77 ± 0.02	77.0 ± 2.0
F2-S	65 ± 3.0	0.26	1.83 ± 0.16	0.25 ± 0.02	−43.0 ± 2.0	0.23 ± 0.03	23.0 ± 2.5
F3-S	45 ± 4.0	0.18	4.50 ± 0.30	0.19 ± 0.03	−42.4 ± 2.3	0.17 ± 0.02	17.0 ± 3.0
F1-V	95 ± 5.0	0.63	1.81 ± 0.17	0.29 ± 0.01	−38.9 ± 2.5	0.18 ± 0.01	90.0 ± 8.0
F2-V	70 ± 3.0	0.28	1.83 ± 0.16	0.5 ± 0.02	−39.9 ± 3.0	0.17 ± 0.02	84.0 ± 4.0
F3-V	58 ± 2.0	0.23	4.50 ± 0.45	0.19 ± 0.03	−40.9 ± 3.2	0.12 ± 0.02	62.0 ± 3.0
F1-T	97 ± 2.0	0.64	1.80 ± 0.15	0.39 ± 0.01	−38.0 ± 3.0	0.25 ± 0.03	125.0 ± 1.5
F2-T	90 ± 1.0	0.36	1.83 ± 0.16	0.3 ± 0.02	−37.9 ± 2.6	0.19 ± 0.02	96.0 ± 5.0
F3-T	82 ± 3.0	0.32	4.50 ± 0.22	0.19 ± 0.03	−37.7 ± 2.6	0.23 ± 0.02	110.0 ± 4.0

**Table 2:** Release kinetics of F1-T proniosomal gel.

	Zero	First	Second	Higuchi	Hixon	Baker
a	21.67742	21.67742	21.67742	2.938152	21.67742	0.020172
b	0.727398	0.727398	0.727398	56.40955	0.727398	0.104645
r	0.797727	−0.79773	0.797727	0.956945	0.797727	0.904567
k	0.727398	−1.6752	0.727398	56.40955	0.727398	0.104645
t (1/2)	68.73817	−0.41368	0.013748	0.78566	1.314504	0.525584

**Table 3:** Composition of the prepared proniosomes loaded with sildenafil, tadalafil, and vardenafil per 2 mL of hydroalcoholic solution containing 0.01% *w*/*v* Pluronic F127.

Formulation Code	Sildenafil (mg)	Tadalafil (mg)	Vardenafil (mg)	Span 60 (mg)	Lecithin (mg)	Cholesterol (mg)	Compritol 888 ATO (mg)	Precirol ATO 5 (mg)
F1-S	20	-	-	400	100	50	-	-
F2-S	20	-	-	300	100	50	100	-
F3-S	20	-	-	300	100	50	-	100
F1-T	-	4	-	400	100	50	-	-
F2-T	-	4	-	300	100	50	100	-
F3-T	-	4	-	300	100	50	-	100
F1-V	-	-	4	400	100	50	-	-
F2-V	-	-	4	300	100	50	100	-
F3-V	-	-	4	300	100	50	-	100

## Data Availability

Data are available upon request.
